# β-1,3-Glucan-Induced Host Phospholipase D Activation Is Involved in *Aspergillus fumigatus* Internalization into Type II Human Pneumocyte A549 Cells

**DOI:** 10.1371/journal.pone.0021468

**Published:** 2011-07-08

**Authors:** Xuelin Han, Rentao Yu, Dongyu Zhen, Sha Tao, Martina Schmidt, Li Han

**Affiliations:** 1 Department for Hospital Infection Control and Research, Institute of Disease Control and Prevention of PLA, Academy of Military Medical Sciences, Beijing, China; 2 Department Molecular Pharmacology, University of Groningen, Groningen, The Netherlands; Montana State University, United States of America

## Abstract

The internalization of *Aspergillus fumigatus* into lung epithelial cells is a process that depends on host cell actin dynamics. The host membrane phosphatidylcholine cleavage driven by phospholipase D (PLD) is closely related to cellular actin dynamics. However, little is known about the impact of PLD on *A. fumigatus* internalization into lung epithelial cells. Here, we report that once germinated, *A. fumigatus* conidia were able to stimulate host PLD activity and internalize more efficiently in A549 cells without altering PLD expression. The internalization of *A. fumigatus* in A549 cells was suppressed by the downregulation of host cell PLD using chemical inhibitors or siRNA interference. The heat-killed swollen conidia, but not the resting conidia, were able to activate host PLD. Further, β-1,3-glucan, the core component of the conidial cell wall, stimulated host PLD activity. This PLD activation and conidia internalization were inhibited by anti-dectin-1 antibody. Indeed, dectin-1, a β-1,3-glucan receptor, was expressed in A549 cells, and its expression profile was not altered by conidial stimulation. Finally, host cell PLD1 and PLD2 accompanied *A. fumigatus* conidia during internalization. Our data indicate that host cell PLD activity induced by β-1,3-glucan on the surface of germinated conidia is important for the efficient internalization of *A. fumigatus* into A549 lung epithelial cells.

## Introduction

*Aspergillus fumigatus (A. fumigatus)* is an airborne fungal pathogen that is known to cause allergic bronchopulmonary aspergillosis, aspergilloma, and invasive aspergillosis [1]. It is now recognized that respiratory epithelial cells provide a surface for host-pathogen interaction and play an important role in the innate defense against pathogenic fungi rather than just acting as a physical barrier [2], [3]. Like many intracellular bacterial pathogens [4], inhaled *A. fumigatus* conidia can bind to lung type II alveolar epithelial cells and invade the cells by inducing their own internalization. Consequently, *A. fumigatus* conidia survive and disseminate within these normally non-phagocytic host cells upon evasion of host defense by phagocytes [5]–[7]. To date, it has been shown that the internalization of *A. fumigatus* conidia into type II A549 lung epithelial cells is closely related to the host cell cytoskeletal dynamics, which induce the invagination of the host cell membrane and the engulfing of the conidia by pseudopods [5], [8], [9].

*A. fumigatus* conidium consists of an outer proteinaceous rodlet layer [10] and an inner cell wall containing several carbohydrate polymers: β-1,3-glucan with β-1,6 branches, linear β-1,3/β-1,4-glucans, galactomannan, and chitin [11]–[13]. The core component of the *A. fumigatus* cell wall is β-1,3-glucan. It is generally accepted that the recognition and induction of inflammatory responses to *A. fumigatus* by host alveolar macrophages rely on the obligate stage-specific exposure of β-1,3-glucan during conidial germination [14]–[17], which is characterized by conidial swelling, dissolution of the rodlet layer, and appearance of polysaccharide moieties on the cell wall [18]. Dectin-1, a major mammalian receptor for β-1,3-glucan is an archetypical non-toll-like pattern recognition receptor that is expressed predominantly by myeloid cells [19]–[21]. Mammalian toll-like receptors (TLR) [22], [23], mannose receptors [24], [25], and complement receptor 3 (CR3) [26], [27] have all been implicated in the recognition of the cell wall components of *A. fumigatus* conidia and hyphae. Thus, recognition and response to *A. fumigatus* may be distinct and variable depending on different host cell types. For instance, the phagocytosis of *A. fumigatus* conidia by murine macrophages involves recognition by dectin-1 and TLR2 [15], [28], whereas CR3 controls the phagocytosis of β-1,3-glucan-bearing particles into human neutrophils [29]. However, the mechanism of *A. fumigatus* internalization into type II lung epithelial cells, specifically the conidial surface molecules and cognate host cell receptors that induce the internalization are presently unknown.

Phospholipase D (PLD) is an important cellular signal modulator that catalyzes the hydrolysis of the most abundant membrane protein phospholipid phosphatidylcholine (PC) to produce phosphatidic acid (PA) and choline. Two mammalian PLD isoforms, PLD1 and PLD2, have been identified thus far. The stimulation of PLD has been described in many cellular systems in response to a large variety of agonist-activated tyrosine kinase receptors and receptors coupled to heterotrimeric G proteins [30], [31]. In mammalian cells, PLD activity is associated closely with actin dynamics. The PLD reaction product, PA, might induce stress fiber formation in suitable cell types [32]–[36] and activate the production of phosphatidylinositol-4,5-bisphosphate (PIP_2_), a significant regulator of actin dynamics [37]. PLD is recognized as an effector of small GTPases (Rho, Rac, and Cdc42) and cofilin, which are all central regulators of cellular actin dynamics [34], [38]–[40]. On the other hand, elements of the actin dynamic system, like β-actin and the actin-binding protein α-actinin, have been demonstrated to influence PLD activity [41], [42]. PLD has also been implicated in the signaling pathway by the Fcγ receptor and the complement receptor [43]–[46]. Essentially, PLD influences the internalization of the facultative intracellular pathogen *Mycobacterium tuberculosis* [47], and plays a critical role in the localized actin dynamic changes in phagocytosis driven by stimulation of the Fcγ receptor [48], [48]–[51]. Moreover, it has been demonstrated that macrophage phagocytosis might be coordinately regulated by PLD1 and PLD2 [37], [52]–[56].

Therefore, we hypothesized that PLD might play an important role in *A. fumigatus* conidia internalization into A549 cells. In the present study, we found that PLD activation induced by *A. fumigatus* β-1,3-glucan through the cellular dectin-1 receptor is potentially a critical step for the efficient internalization of *A. fumigatus* into respiratory epithelial cells.

## Results

### 1. *Aspergillus fumigatus* induces host PLD activation during its internalization into A549 cells

**Figure 1:**
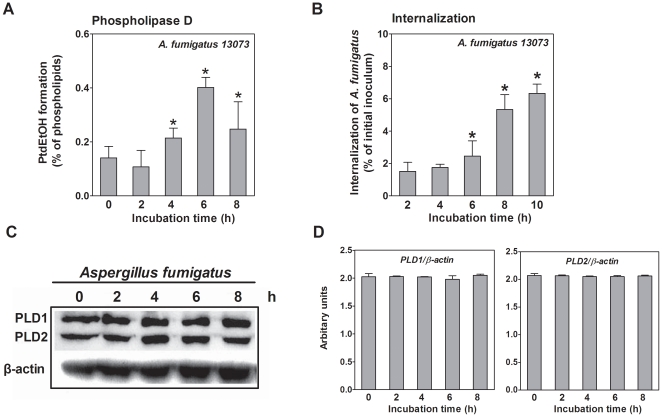
*A. fumigatus* stimulates PLD activity during its internalization into A549 cells. A. A549 cells were prelabeled with [^3^H] oleic acid and infected with the resting conidia of *A. fumigatus* 13073 at an MOI of 10 for the indicated time periods. Then, ethanol was added to determine the PLD activity. B. A549 cells were infected with the resting conidia of *A. fumigatus* 13073 at an MOI of 10 for the indicated time periods, and the internalization of *A. fumigatus* was analyzed by the nystatin protection assay. Differences in [^3^H] PtdEtOH formation between the 0 h time point and the other time points (A) and differences in the internalization of *A. fumigatus* between the 2 h time point and the other time points (B) were compared. In parallel, the cells were lysed for immunoblotting with the indicated antibody (C) and the densitometric analysis of immunoblots for three independent experiments is shown (D). Data are represented as the mean ± SE (n = 3–4), and the blots are characteristic of 3 independent experiments. *P<0.05.

First, we studied the alterations in host cell PLD activity during the internalization of *A. fumigatus* into A549 epithelial cells. The cells were infected with the live resting conidia of *A. fumigatus* at a multiplicity of infection (MOI) of 10 (conidia: cells) in the absence of serum. As illustrated in Figure 1A, PLD activity was rarely detectable during the first 2 h of interaction between the conidia and the cells. However, incubations ≥4 h largely enhanced host cell PLD activity by approximately 3-fold, persisting for up to 8 h of co-incubation (Figure 1A). In parallel, during the first 4 h of co-incubation, the internalization of *A. fumigatus* conidia did not change tremendously. Beyond 4 h, the internalization of *A. fumigatus* conidia increased in a time-dependant manner, reaching a level of approximately 160% after 6 h and approximately 3-fold after 8 h (Figure 1B), which was consistent with the time-curve of host cell PLD activity. The endogenous expression of PLD1 and PLD2 was not affected during the co-incubation process (Figure 1C and 1D). These data suggest that host cell PLD activity is stimulated by the interaction of *A. fumigatus* conidia with A549 cells, and the induction of PLD activity might be related to the internalization of *A. fumigatus* conidia.

### 2. Induction of PLD activation in A549 cells by *A. fumigatus* requires conidial germination

**Figure 2:**
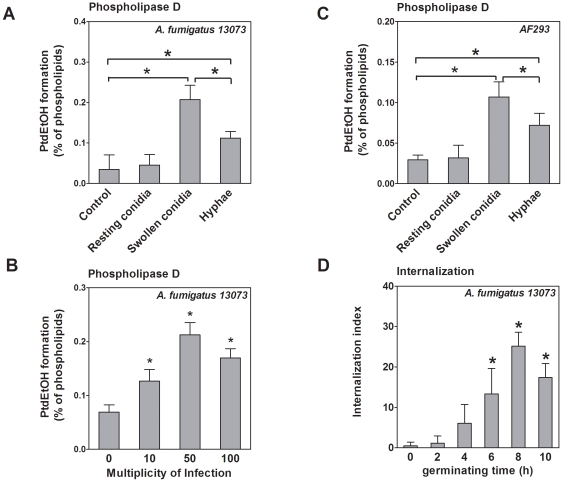
Host cell PLD activity is stimulated by swollen conidia, but not resting conidia. **A.** A549 cells were infected with the live resting conidia, swollen conidia (germinated for 6 h), and hyphae (germinated for 12 h) of *A. fumigatus* 13073 at an MOI of 10 for 30 min. B. A549 cells were infected with the swollen conidia of *A. fumigatus* 13073 for 30 min at the indicated MOI. C. A549 cells were infected with live resting conidia, swollen conidia and hyphae of *A. fumigatus* AF293 at an MOI of 10 for 30 min. Thereafter, the PLD activity in the A549 cells was measured, and the differences in [^3^H] PtdEtOH formation between uninfected group (Control) and infected group or between groups were compared as indicated in the figure (A, B, C). D. A549 cells were infected with resting conidia (germinating time = 0), or conidia of *A. fumigatus* 13073 germinated for the indicated time periods at an MOI of 10 for 60 min. *A. fumigatus* internalization was determined by immunofluorescent staining. Differences in the internalization index between resting conidia and germinated conidia were compared. Data are represented as the mean ± SE (n = 3–4). *P<0.05.

The resting conidia of *A. fumigatus* swell after 6 h in a suitable environment [8]. Thus, we checked the conidial shape microscopically and found a positive correlation between the time of co-incubation and the percentage of swollen conidia, which reached 80% after 6 h. The germination of *A. fumigatus* conidia is a complicated biological process and induces various host cell responses. We hypothesized that the induction of host cell PLD activity during co-incubation with *A. fumigatus* conidia might be related to conidial germination. Therefore, we compared the host cell PLD activity induced with resting conidia, swollen conidia (germination), and the hyphae of *A. fumigatus* 13073. As shown in Figure 2A, the resting conidia did not increase host cell PLD activity, whereas swollen conidia and hyphae increased PLD activity by approximately 4- and 3-fold, respectively. The hyphae were less potent in inducing PLD activation than the swollen conidia (Figure 2A). In addition, induction of host cell PLD activation by swollen conidia occurred in a concentration-dependent manner and reached its maximum at an MOI of 50 (Figure 2B). The swollen conidia and hyphae of the *A. fumigatus* strain AF293, but not the resting conidia, also increased host cell PLD activity, indicating that the germination-dependent induction of PLD activation is not strain-specific (Figure 2C). To determine alterations in the internalization of *A. fumigatus* conidia during germination, we compared the internalization index of resting conidia with that of conidia germinated for different times using the immunofluorescence assay. The internalization index of germinated *A. fumigatus* conidia increased significantly in a time-dependent manner. The internalization index of *A. fumigatus* conidia germinated for 6 h (13.3%±6%) was approximately 26-fold higher than that of resting conidia, (0.51%±0.9%; Figure 2D). These data indicate that conidial germination induces host cell PLD activation and enhances *A. fumigatus* conidia internalization into lung epithelial cells.

### 3. PLD inhibitors suppress the internalization of *A. fumigatus* into A549 cells

**Figure 3:**
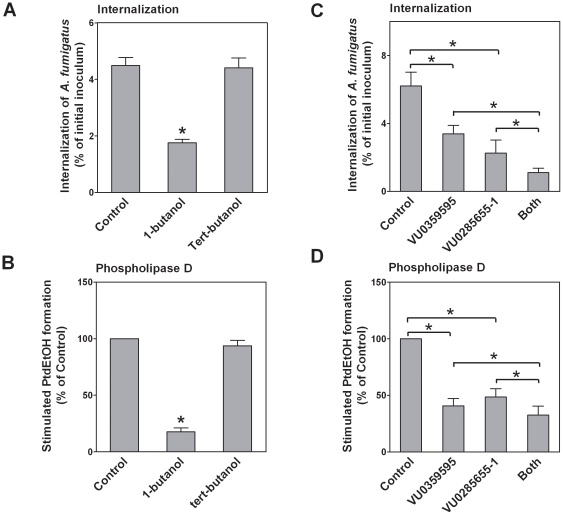
Inhibition of *A. fumigatus* internalization by PLD chemical inhibitors. A549 cells were incubated for 30 min with 1% (v/v) 1-butanol or tert-butanol (A, B), 2 nM VU0359595 (PLD1-specific inhibitor), 100 nM VU0285655-1 (PLD2-specific inhibitor), or both (C, D). Subsequently, the cells were infected with *A. fumigatus* 13073 swollen conidia at an MOI of 10. *A. fumigatus* internalization was analyzed by the nystatin protection assay (A, C) and PLD activities (B, D) were measured. Differences in [^3^H] PtdEtOH formation or *A. fumigatus* internalization between the untreated (control) cells and inhibitor-pretreated cells were compared. Data are represented as the mean ± SE (n = 3–4). *P<0.05.

It has been reported that the inhibition of PLD activity might impair macrophage phagocytosis [46], [47]. We investigated the role of host cell PLD activity on *A. fumigatus* internalization into A549 cells. In the presence of primary alcohols, like 1-butanol, PLD transfers the phosphatidyl groups from PC and phosphatidylethanolamine to the alcohol, generating phosphatidylbutanol rather than its normal product PA. Therefore, 1-butanol is often used as PLD-non-specific inhibitor [31]. In contrast, tert-butanol does not competitively alter PLD activity and is usually used as control for 1-butanol. Pretreatment of the A549 cells with 1% (v/v) 1-butanol inhibited the internalization of swollen conidia by approximately 59.8±9.2% (Figure 3A). Likewise, 1-butanol largely reduced conidia-induced host cell PLD activation (Figure 3B). In contrast, tert-butanol did not reduce conidia-induced PLD activation and conidia internalization (Figure 3A and 3B). Pretreatment of A549 cells with the PLD1-specific inhibitor VU0359595 reduced *A. fumigatus* internalization (Figure 3C) and conidia-induced PLD activation (Figure 3D) by approximately 50%. Similar results were also found in A549 cells pretreated with the PLD2-specific inhibitor VU0285655-1 (Figures 3C and 3D). The inhibitor concentrations did not compromise A549 cell viability as determined by crystal violet staining (data not shown). Interestingly, the combination of both PLD inhibitors further enhanced the inhibition of conidia internalization (Figure 3C) and host cell PLD activity (Figure 3D). These data suggest that both PLD1 and PLD2 host cell activities are required for efficient *A. fumigatus* internalization into lung epithelial cells.

### 4. Silencing of endogenous host cell PLD expression suppresses internalization of *A. fumigatus* into A549 cells

**Figure 4:**
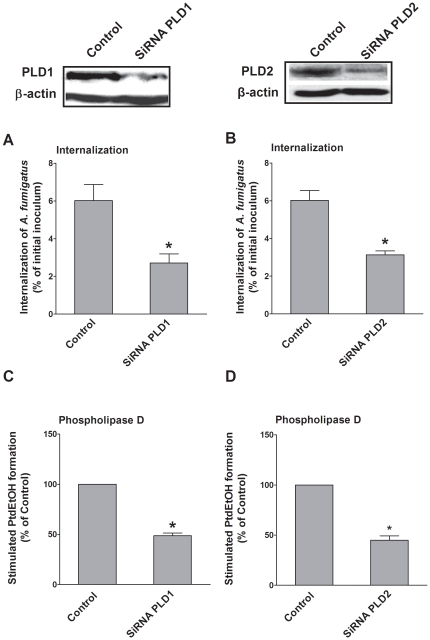
Interference of endogenous PLD host cell expression reduces the internalization of *A. fumigatus*. A549 cells were transfected with non-specific small interfering RNAs (siRNAs) (Control), PLD1-specific siRNAs (A, C), or PLD2-specific siRNAs (B, D). After 48 h, cells were infected with the swollen conidia of *A. fumigatus* 13073 at an MOI of 10. *A. fumigatus* internalization was analyzed by the nystatin protection assay (A, B) and PLD activities (C, D) were measured. Differences in [^3^H] PtdEtOH formation and *A. fumigatus* internalization between the untransfected cells (Control) and PLD-silenced cells were compared. Data are represented as the mean ± SE (n = 3–4). *P<0.05. The immunoblots represent PLD1 and PLD2 expression in A549 cell lysates.

To directly establish whether endogenous PLD is involved in *A. fumigatus* internalization, we reduced the expression of endogenous PLD1 and PLD2 with small interfering RNAs (siRNAs) as illustrated in Figure 4. The internalization of swollen conidia into A549 cells expressing siRNAs targeting either PLD1 or PLD2 was diminished by approximately 50% as compared with cells transfected with control siRNA (Figures 4A and 4B). Likewise, conidia-induced host cell PLD activity was also inhibited by either PLD1 or PLD2 silencing by approximately 50% (Figures 4C and 4D). Because co-silencing of PLD1 and PLD2 compromised A549 cell viability, we did not investigate their synergistic effect on internalization. Taken together, our data indicate that both PLD1 and PLD2 are indispensable for *A. fumigatus* internalization into A549 cells.

### 5. Heat-killed swollen conidia stimulate PLD activity and internalize into A549 cells

**Figure 5:**
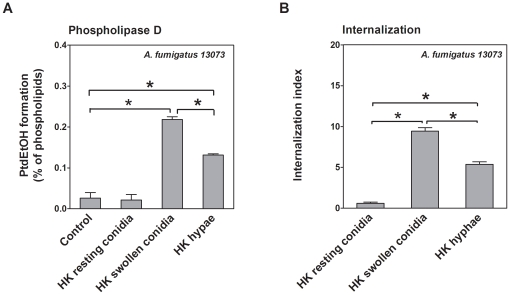
Heat-killed swollen conidia stimulate PLD activity and internalize in A549 cells. A549 cells were infected with heat-killed (HK) resting conidia, HK swollen conidia (germinated for 6 h), and HK hyphae (germinated for 12 h) of *A. fumigatus* 13073 (at an MOI of 10 each). The PLD activities were measured (A) and *A. fumigatus* internalization was determined by immunofluorescent staining (B). Differences in [^3^H] PtdEtOH formation and in the internalization indices between groups were compared as indicated in the figure. Data are represented as the mean ± SE (n = 3–4). *P<0.05.

We intended to identify the conidial component involved in the induction of host cell PLD activity and conidia internalization. Heat killing is known to inactivate conidial surface proteins and to stop conidial germination at defined time points [14]; therefore, we investigated the effect of heat-killed conidia or hyphae on host cell PLD activity and conidia internalization. Heat-killed swollen conidia and hyphae, but not heat-killed resting conidia, strongly induced host cell PLD activity in a manner similar to live conidia (Figure 5A). In parallel, the internalization of heat-killed swollen conidia of *A. fumigatus* 13073 into A549 cells increased as compared with heat-killed resting conidia (Figure 5B). However, the heat-killed hyphae were less potent to induce host cell PLD activation and conidia internalization, as compared with the heat-killed swollen conidia (Figure 5A and 5B). Thus, our data suggest that some heat-insensitive components on the germinated conidial surfaces are responsible for the induction of the PLD response in A549 cells upon conidia internalization.

### 6. β-1,3-glucan induces PLD activation in A549 cells

**Figure 6:**
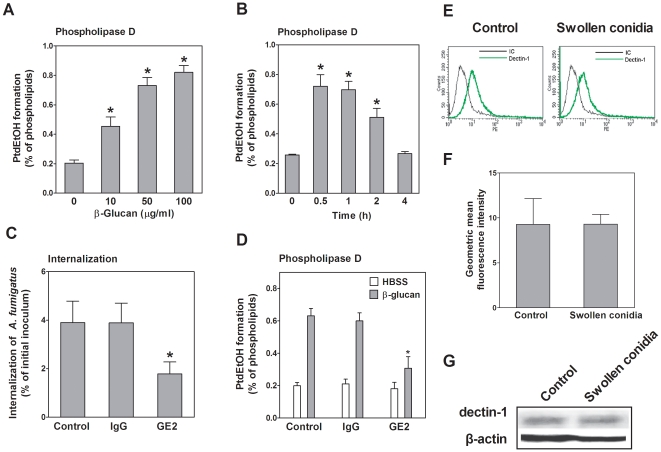
β-1,3-glucan induces PLD activity in A549 cells. A549 cells were stimulated with the indicated concentrations of β-1,3-glucan for 30 min (A) or for the indicated duration of time with 50 µg/mL of β-1,3-glucan (B). Thereafter, the PLD activity was determined and differences in [^3^H] PtdEtOH formation between the control (indicated by “0”) and other groups were compared. In C and D, A549 cells were first incubated with HBSS (Control), HBSS containing 5 µg/mL of isotype control antibody and HBSS containing 5 µg/mL of anti-dectin-1 mAb GE2 (ab82888) for 30 min, respectively. Then, the cells were infected with swollen conidia of *A. fumigatus* 13073 at an MOI of 10 (C) or stimulated by HBSS and HBSS containing 50 µg/mL of β-1,3-glucan for 30 min, respectively (D). *A. fumigatus* internalization was analyzed by the nystatin protection assay (C) and the PLD activity (D) was measured. Differences in *A. fumigatus* internalization and [^3^H] PtdEtOH formation between the untreated cells (Control) and antibody-treated cells were compared. In E and F, A549 cells were infected with swollen conidia of *A. fumigatus* 13073 at an MOI of 10 or incubated with PBS (Control) for 30 min. Subsequently, the cells were stained with isotype (IC) antibody or the primary anti-dectin-1 mAb GE2 (ab82888) and analyzed by FACS Calibur flow cytometer. The geometric mean fluorescence intensity was determined by Cell Quest Pro software. Differences between uninfected cells (Control) and conidia-infected cells were compared. G. A549 cells were incubated with swollen conidia of *A. fumigatus* 13073 at an MOI of 10 or incubated with PBS (Control) for 30 min. Cells were analyzed for dectin-1 expression by immunoblotting using an anti-dectin-1 antibody (sc-26094). FACS profiles and immunoblots shown here are characteristic of 3 independent experiments. Data are represented as mean ± SE (n = 3–4). *P<0.05.

*A. fumigatus* conidial swelling was accompanied by surface stage-specific exposure of β-1,3-glucan polymers [14] and a heat-insensitive component seemed to mediate conidia-induced PLD activity in A549 cells (see above). We hypothesized that β-1,3-glucan polymers may be the agonist to induce PLD activity and to regulate the internalization process of *A. fumigatus* conidia. To test this hypothesis, we first used β-1,3-glucan polymers to stimulate A549 cells at increasing concentrations. Strikingly, host cell PLD activity increased in a concentration-dependent manner, reaching its maximum of approximately 4-fold upon addition of 50–100 µg/mL β-1,3-glucan (Figure 6A). Consequently, the stimulation of A549 cells with 50-µg/mL β-1,3-glucan increased host cell PLD activity, reaching its maximum after 0.5–1 h incubation (Figure 6B). Next, we tested whether the β-1,3-glucan-induced host cell PLD activation might also alter *A. fumigatus* internalization. Because dectin-1 is the major receptor for β-1,3-glucan, the monoclonal GE2 antibody (mAb GE2) against dectin-1 was used to block the dectin-1 receptor. This treatment reduced *A. fumigatus* internalization by approximately 50% (Figure 6C). In parallel, β-1,3-glucan-induced host cell PLD activation was largely reduced by pretreatment with mAb GE2, but not with the IgG isotype control antibody (Figures 6C and 6D). In addition, because dectin-1 is predominately expressed by myeloid cells [20], [21], we investigated the expression of dectin-1 on A549 cells using flow cytometry. As shown in Figures 6E and 6F, dectin-1 was indeed expressed in resting A549 cells, and the swollen conidia did not upregulate dectin-1 expression on A549 cells as compared with the non-stimulated cells. These results were confirmed by immunoblot analysis using an anti-dectin-1 antibody (Figure 6G). Taken together, these data indicated that the β-1,3-glucan on the surface of germinated conidia induced host cell PLD activity most likely upon interaction with the dectin-1 receptor.

### 7. PLD accompanies conidia during *A. fumigatus* internalization into A549 cells

**Figure 7:**
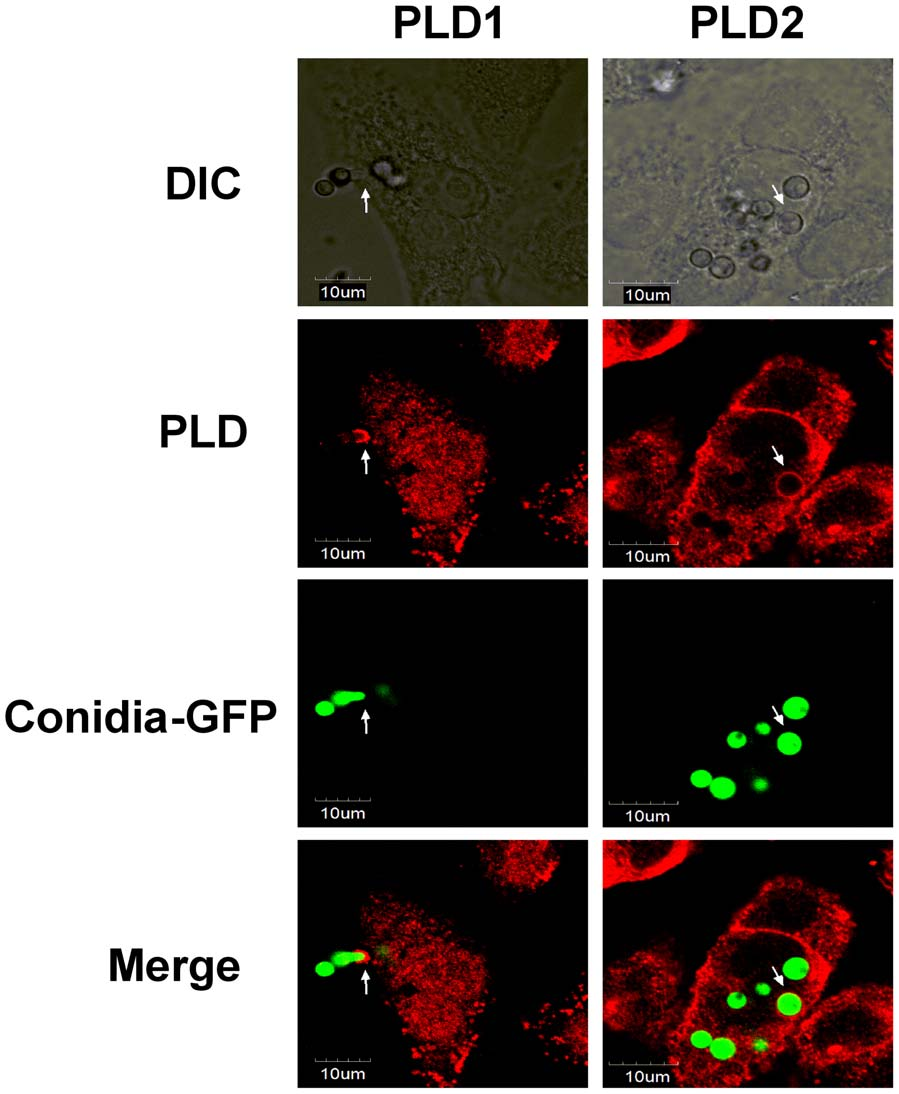
Both PLD1 and PLD2 are associated with *A. fumigatus* conidia during internalization into A549 cells. A549 cells were infected with the swollen conidia of *A. fumigatus* 13073 stably expressing green fluorescence protein (at an MOI of 10) for 30 min. The internalization of *A. fumigatus* into A549 cells was monitored by differential interference contrast (DIC) microscopy or immunofluorescence using laser confocal microscopy Olympus FluoView FV1000 (green, *A. fumigatus* conidia; red, PLD1 or PLD2). The images were processed with Olympus FluoView ver. 1.6., and the merged fluorescence images are shown. The data represent 3 similar experiments, and the arrows (white) indicate the association of internalized conidia with PLD. Scale bar, 10 µm.

It has been previously reported that PLD plays a critical role in the changes in localized actin dynamics driven by the Fcγ receptor during phagocytosis [46], [48]–[50]. The distribution of PLD during *A. fumigatus* conidia internalization was investigated by confocal microscopy. The invasion (internalization) of the green-fluorescent-labeled germinating conidia of *A. fumigatus* 13073 into A549 cells along with a red-labeled PLD1 cap at the invasion site is shown in Figure 7 (left panel, arrow); PLD2 was enriched around an internalized conidium and thereby formed a red-labeled ring (Figure 7, right panel, arrow). In addition, PLD1 was primarily localized in the intracellular compartments and the cytosol, whereas PLD2 was primarily localized at the plasma membrane (Figure 7). These results were consistent with previous data from several cell types [57]–[59]. Taken together, the images indicate that both PLD1 and PLD2 associate with *A. fumigatus* conidia during the internalization process into A549 cells.

## Discussion

PLD is an essential regulator of cellular glycerophospholipid metabolism [60], [61]. Indeed, receptor-controlled PLD activity is involved in macrophage phagocytosis mainly through localized the accumulation of PA or direct interaction with regulatory molecules [31], [46], [48], [53], [62], [63]. However, there is limited data on the role of PLD in pathogen-induced internalization into non-phagocytic cells, such as epithelial and endothelial cells. Our findings that *A. fumigatus* internalization into A549 lung epithelial cells induces PLD activity are in agreement with the previously published observation that the phagocytosis of *M. tuberculosis* and opsonized zymosan is tightly coupled to PLD activity in human macrophages [47], [53]. Similarly, it has been reported that host PLD activity in J774 murine macrophage-derived cells is also enhanced upon infection with the intracellular pathogen *Listeria monocytogenes* [64]. Further, isoform-specific inhibitors and RNA interference are accepted as reliable tools to assess PLD functions in many physiological processes [64]–[67]. The clear reduction of *A. fumigatus* internalization into A549 cells upon downregulation of endogenous PLD1 or PLD2 perfectly reflects the putative role of PLD in Fcγ receptor-mediated phagocytosis [46], [48] and receptor internalization [68], [69]. Nevertheless, due to our serum-free incubation, it is reasonable to assume that *A. fumigatus*-induced PLD activity in A549 epithelial cells is not Fcγ- or complement-mediated. Further, both PLD1 and PLD2 were localized on swollen conidia that were already internalized or still in the process of internalization (Figure 7). This phenomenon is slightly different from the data obtained in macrophages that revealed that PLD1-bound vesicles might predominantly exist in the phase of phagosome maturation, whereas PLD2 usually correlated with the formation of nascent phagosomes during IgG-opsonized phagocytosis by macrophages [48]. We observed a clear red-labeled PLD1 cap at the side of invading conidia and a red-labeled PLD2 ring at the side of the internalized conidia. Thus, our data indicate that, most likely through direct interaction, both PLD1 and PLD2 might play an essential role in phagosome formation and development during the internalization of *A. fumigatus* into A549 lung epithelial cells. Certainly, a more subtle spatial or temporal relationship between conidia and PLD enzymes during the internalization should be investigated in future studies.

Another important finding in the present study is that conidial germination is tightly coupled with both host cell PLD activity and *A. fumigatus* internalization, indicating the involvement of β-1,3-glucan in both processes. Indeed, earlier studies suggest that β-1,3-glucan is exposed on the conidial surfaces in a stage-specific fashion [14], [15], and *A. fumigatus* swollen conidia or early germlings with high amounts of surface-exposed β-1,3-glucan are able to trigger strong inflammatory responses by activation of the dectin-1 receptor [16]. Consistent with these findings, swollen conidia and early germlings of *A. fumigatus* induced host cell PLD activation and exhibited an increased internalization capacity as compared with resting conidia. Likewise, it has been shown that exposure of β-1,3-glucan increased *A. fumigatus* conidia phagocytosis by macrophages [15]. The significant difference between swollen conidia and hyphae in inducing host PLD activation is consistent with two previous studies, which showed that beta-glucan moieties exposed on mature hyphae were present and recognized by specific antibodies at a much lower intensity than swollen conidia [16], [70]. Heat-killed swollen conidia that were inactivated after the initiation of the germination process largely increased host cell PLD activity and were still taken up by A549 cells, which indicates that the induction of host cell PLD activity and conidia internalization are most likely related to some heat-stable components exposed on the surface of conidia. However, autoclaving can strip substances from the conidial cell wall and expose moieties that may not be exposed on the surface of viable conidia. Thus, the mechanism of the induction of PLD activation and uptake of autoclaved conidia may be different from that of viable conidia. We also generated a pigment-deficient white mutant of *A. fumigatus* 13073 with UV mutation. The swollen conidia of this mutant could induce host cell PLD activation as well (data not shown), which indicates, to some extent, that *A. fumigatus*-induced PLD activity in A549 cells might be not associated with conidial pigments, although the loss of pigment by UV mutagenesis is not as defined as the pigment-deficiency caused by the deletion in the *PksP* pathway. Taken together, it is reasonable to speculate that β-1,3-glucan exposed on the cell wall of *A. fumigatus* might act as an agonist for host cell PLD activity.

Indeed, we found that, similar to swollen conidia, the pure β-1,3-glucan particle alone is capable of inducing host cell PLD activation (Figure 6). This data indicate that a cellular receptor for β-1,3-glucan might mediate PLD activation. It is known that dectin-1 is the mammalian receptor for the β-1,3-glucan-mediated phagocytosis of many fungal pathogens by macrophages and leukocytes; however, the human dectin-1 is predominantly expressed in B cells, mast cells [71], [72] and myeloid cells, including macrophages, dendritic cells, monocytes, neutrophils, and a subset of splenic T cells [21]. So far, a limited number of reports indicate expression of dectin-1 on lung epithelial cells and hint to a potential role of dectin-1 in *A. fumigatus* internalization into lung epithelial cells. However, a few recent studies have indicated that dectin-1 expression is induced in other non-leukocytes, such as bronchiolar epithelial cells and alveolar type II cells of lungs exposed to curdlan (a kind of β-1,3-glucan) [73], human bronchial epithelial cells challenged by Poly IC [74], epidermal keratinocytes by *Mycobacterium ulcerans*, and A549 cells by *Mycobacterium tuberculosis* [75], [76]. Here, we report that the anti-dectin-1 mAb GE2 suppressed swollen conidia- or β-1,3-glucan-induced PLD activity and conidia internalization (Figures 6C and 6D), indicating the existence of dectin-1 in A549 cells. Further, detection of dectin-1 by western blot revealed that dectin-1 is expressed on the surface of A549 cells, regardless of infection by *A. fumigatus* swollen conidia. These results were confirmed by flow cytometry (Figure 6E), suggesting that dectin-1 might be constitutively expressed on A549 cells, and β-1,3-glucan exposed on the outside of germinating conidia probably binds dectin-1 to stimulate PLD activity that results in conidia internalization. Although there might be a discrepancy between our results and the inducible dectin-1 expression observed in previous studies [75], [76], it should be noted that in the previous studies, dectin-1 was detected at 5 min after mycobacterium infection in A549 cells. Moreover, induction of PLD activity by β-1,3-glucan and *A. fumigatus* internalization was not fully blocked by an anti-dectin-1 antibody, indicating that a non-dectin-1 β-1,3-glucan receptor [20] might mediate PLD activation and internalization as well. CR3 might be a candidate receptor because it can recognize β-1,3-glucan [77], [78] and has been shown to regulate the phagocytosis of β-1,3-glucan-bearing particles by human neutrophils [29], although its exact mechanism is not completely clear.

In conclusion, our results demonstrate for the first time that β-1,3-glucan induces PLD activity most likely upon interaction with the dectin-1 receptor, highlighting a novel regulatory component of *A. fumigatus* internalization into epithelial cells. However, further investigations are still required to elucidate the exact molecular mechanism of receptor-mediated PLD activity and its precise function during *A. fumigatus* internalization into lung epithelial cells.

## Materials and Methods

### *A. fumigatus* strains, cell line

*A. fumigatus* ATCC13073 constitutively expressing green fluorescent protein was generously provided by Dr. Margo Moore (Simon Fraser University, Burnaby, BC, Canada) and used for the internalization and PLD activity assays. *A. fumigatus* AF293 was a gift from Dr. KJ. Kwon-Chung (National Institute of Health, Bethesda, Maryland). Except where indicated, all *A. fumigatus* strains were propagated on Sabouraud dextrose agar (10 g/L peptone, 40 g/L glucose, and 15 g/L agar) for 5∼8 days at 37°C. The type II human pneumocyte cell line A549 was obtained from ATCC and cultured in DMEM (GIBCO) supplemented with 10% fetal calf serum, 100 U/mL streptomycin, and 100 U/mL penicillin at 37°C in a humidified 5% CO_2_ incubator.

### Preparation of conidia

*A. fumigatus* conidia were harvested and prepared as described in a previous study [14]. Briefly, after 5∼8 days culture, *A. fumigatus* conidia were dislodged from agar plates by gentle washing and resuspended in sterile phosphate-buffered saline supplemented with 0.1% Tween 20 (PBST). The conidia were then passed through 8 layers of sterile gauze to remove hyphal fragments and enumerated on a hemacytometer. The conidia were incubated at 37°C in DMEM for the indicated times. Preparations incubated for 6 h contained swollen conidia with early germlings with <5 µm hyphal extensions. Preparations incubated for 12 h included hyphae. Fungal cells were washed twice and stored at 4°C for use within 48 h. As required, heat inactivation was performed at 121°C for 15 min in an autoclave.

### Chemical reagents and antibodies

β-1,3-glucan from *Euglena gracilis* (Flucan 89862) was used in the experiments and a fresh stock solution was prepared for each in vitro experiment by dissolving it in pyrogen-free phosphate-buffered saline (PBS). 1-butanol and tert-butanol was purchased from Sigma-Aldrich and [^3^H]oleic acid (33.4 Ci/mmol) was obtained from PerkinElmer, Inc.. The PLD1-specific inhibitor VU0359595 and the PLD2-specific inhibitor VU0285655-1 were purchased from Avanti Polar Lipids. For the immunofluorescence analysis, the rabbit polyclonal anti-PLD1 antibody (sc-25512), goat polyclonal anti-PLD2 antibody (sc-48270), and mouse monoclonal anti-β-actin antibody (sc-47778) were obtained from Santa Cruz Biotechnology Inc. The secondary antibody TRITC-conjugated goat anti-rabbit IgG was purchased from Southern Biotech, while TRITC-conjugated rabbit anti-goat IgG was obtained from ZSGB-BIO, China. For the western blot analysis, the rabbit polyclonal anti-PC-PLD1 (44–320) and rabbit polyclonal anti-PLD2 (44–326) antibodies were purchased from Invitrogen. The rabbit anti-PLD1-41 and rabbit anti-PLD2-41 antibodies were gifts from Dr. Sylvain Bourgoin, Canada. The goat polyclonal antibody against dectin-1 (sc-26094) was obtained from Santa Cruz Biotechnology Inc. HRP-conjugated goat anti-mouse IgG and HRP-conjugated goat anti-rabbit IgG antibodies were obtained from ZSGB-BIO, China. For blocking and flow cytometric analysis, anti-dectin-1 mAb GE2 (ab82888) was commercially available from Abcam Inc., and the isotype control mAb (IgG1) was purchased from eBioscience. PE-conjugated goat anti-mouse IgG was obtained from Multiscience Biotech.

For preparation of the anti-*Aspergillus* antibody, a suspension containing 1×10^7^ *A. fumigatus* ATCC13073 conidia/mL was heated at 121°C for 15 min and emulsified with an equal volume of Freund's complete adjuvant. A two-milliliter mixture was injected subcutaneously into each of the 2 male New Zealand white rabbits. Fourteen days later, the rabbits were injected with the same dose of the above mixture. To test the serum for the presence of antibodies that recognize *A. fumigatus*, conidia from strain 13073 were added to 12-mm-diameter number 1 coverslips in 24-well plates and reacted with various dilutions of serum prepared in PBS-1% (w/v) bovine serum albumin (BSA). The bound primary antibody was detected by a goat anti-rabbit TRITC-conjugated secondary antibody diluted 1∶100 in PBS-1% BSA. Conidia were fixed with 4% (w/v) paraformaldehyde (PFA) in PBS and viewed with an Olympus BX51 microscope equipped for epifluorescence microscopy.

### RNA interference

For PLD interference, pairs of 21-nucleotide sense and antisense RNA oligomers were designed and chemically synthesized by Sigma-Aldrich. The oligonucleotides for PLD1 were as follows: sense, 5′-CAA GAG AAU GCU UUA GCU A[dT][dT]-3′ and antisense, 5′-UAG CUA AAG CAU UCU CUU G[dT][dT]-3′ (SASI_Hs01_00239086). The oligonucleotides for PLD2 were as follows: sense, 5′-UAU GAG CAG AUC UUC CGC U[dT][dT]-3′ and antisense, 5′-AGC GGA AGA UCU GCU CAU A-3′ (SASI_Hs01_00113016). The siRNA universal negative control #1 (Sigma) was used. For transfection, the cells were plated in 35-mm dishes and transfected with 3.5 µL X-tremeGENE siRNA Transfection Reagent (Roche) in 100 µL Opti-MEM containing 2 µg of PLD1 or PLD2 siRNA.

### Analysis of *A. fumigatus* internalization

The nystatin protection and immunofluorescence assays to determine the *A. fumigatus* internalization into A549 cells were performed as previously described, and both techniques were well correlated in determining the internalization of *A. fumigatus* into A549 cells [8]. For the nystatin protection assay, A549 cells pretreated with non-specific PLD inhibitors, antibodies, or transfected by different plasmids were seeded at 2×10^4^ cells/well in 96-well plates (Corning) and grown for 16 h. The cells were incubated with *A. fumigatus* conidia at the indicated MOI under centrifugation at 200 *g* for 5 min at 4°C. Internalization was performed by incubation at 37°C for the indicated times in 5% CO_2_. Subsequently, the wells were washed 3 times with PBST and incubated with nystatin (20 µg/mL) in DMEM for 3 h at 37°C. The monolayers were washed twice with PBST and lysed by incubating in 0.25% Triton X-100 for 15 min. The released conidia were diluted and plated onto SDA agar (3 replicate plates/well) and incubated at 37°C for 24 h. The colonies were counted to determine the total intracellular conidia. The internalization capacity is expressed as a percentage of the initial inoculum.

For the immunofluorescence assay, A549 cells were seeded on 12-mm-diameter coverslips in 24-well plates at 1×10^5^ cells/well (Corning) and grown for 16 h. The cells were infected with 1 mL of 10^6^ conidia/mL in DMEM for the indicated times at 37°C. After infection, unbound conidia were removed by washing 3 times with PBST, and the cells were incubated for 1 h in PBS containing 0.1% (w/v) BSA (Roche 738328) at 37°C. Afterwards, extracellular conidia were labeled with the anti-*A. fumigatus* rabbit antibody (1∶20) and goat anti-rabbit TRITC-conjugated secondary antibody (1∶100). Then, the wells were washed and fixed for 15 min with 4% (w/v) PFA (pH 7.4)/PBS. Finally, the coverslips were mounted on a slide and observed under a BX51 fluorescent microscope (Olympus). To analyze the uptake of conidia, 10 fields per coverslip were captured with an Olympus DP71 camera using Image Pro Express (IPE) for image capturing (Media Cybernetics Inc., MA, USA). The extracellular and total conidia (extracellular+intracellular) were enumerated under the red and green channels in IPE, respectively. The number of internalized conidia was calculated by the subtraction of the extracellular conidia from the total conidia. The internalization index determined by the immunofluorescence assay is the number of internalized conidia divided by the number of total conidia per field ×100.

In the present study, the internalization of *A. fumigatus* into A549 cells was analyzed using the nystatin protection assay with the exception of the two experiments in Figure 2D and Figure 5B. In Figure 2D, the immunofluorescence assay was used because the 3-h nystatin incubation step in the nystatin protection assay might interfere with the accurate comparison of the internalization capacity of *A. fumigatus* conidia in different germinating conditions, and the immunofluorescence method is comparatively suitable way to analyze this difference. Indeed, our preliminary data showed that the internalization of resting conidia was usually much higher than that of conidia germinated for 2 h using the nystatin protection assay (data not shown). This phenomenon might result from the fact that nystatin is only fungicidal for germinating conidia and not all resting conidia can be germinated and killed during the 3-h nystatin incubation. In Figure 5B, the immunofluorescence assay was also performed because the nystatin protection assay could only be used to determine the internalization of viable *A. fumigatus* conidia.

### Assay of PLD activity in intact cells

For measurement of cellular PLD activity, the pretreated cells were replated 24 h after transfection onto 35-mm culture dishes. The cellular phospholipids were labeled by incubating the cell monolayer for 20–24 h with [^3^H]oleic acid (2 µCi/mL) in growth medium. Thereafter, the cells were washed twice in Hank's balanced salt solution (HBSS) containing 118 mM NaCl, 5 mM KCl, 1 mM CaCl_2_, 1 mM MgCl_2_, and 5 mM d-glucose buffered at pH 7.4 with 15 mM HEPES. Then, the cells were stimulated by the *A. fumigatus* conidia variant at the indicated MOI or by β-1,3-glucan at the indicated concentration in the presence of 2% ethanol at 37°C for 30 min. Alternatively, the cells were incubated with the resting conidia of the variable *A. fumigatus* in HBSS at 37°C for the indicated time. Thereafter, ethanol was immediately added for an additional 30-min incubation. The reaction was stopped by the addition of ice-cold methanol/chloroform (1∶1, v/v), and labeled phospholipids, including the specific PLD product [^3^H]phosphatidylethanol ([^3^H]PtdEtOH), were isolated as described previously [38]. The formation of [^3^H]PtdEtOH is expressed as a percentage of the total amount of labeled phospholipids.

### Immunofluorescence analysis and immunoblotting

A549 cells (5×10^4^/mL) were seeded onto coverslips in 24-well plates and grown for 24 h. The conidia variants were added to the wells to start the invasion as described above. To stop internalization, the cells were washed once with ice-cold PBS and fixed in ice-cold 4% PFA in PBS buffer for 20 min at room temperature. The cells were then permeabilized for 4 min with 0.05% Triton X-100 in PBS buffer and stained with the indicated antibodies. The primary antibodies used for PLD staining were anti-PLD1 (sc-25512, 1∶100) and anti-PLD2 (sc-48270, 1∶100). The secondary antibodies used for PLD1 were TRITC-conjugated goat anti-rabbit IgGs (1∶50) and for PLD2 were TRITC-conjugated rabbit anti-goat IgGs (1∶50). The preparations were observed with a laser scanning confocal microscope Olympus FluoView FV1000. Green fluorescence was captured with a 515- to 540-nm band pass filter, and red fluorescence was captured with a 590- to 610-nm band pass filter. The images were processed with an Olympus FluoView ver. 1.6.

For immunoblotting, equal amounts of protein from cell lysates were separated by SDS-PAGE and transferred to a PVDF membrane. Then, the membrane was incubated for 3 h with the appropriate primary antibodies and subsequently incubated for 3 h with the HRP-conjugated secondary antibody. The proteins were visualized by enhanced chemiluminescence (Santa Cruz Biotechnology Inc.). A densitometric analysis of immunoblots was performed by BandScan 5.0 (Glyko Inc. Canada).

### Antibody inhibition experiments

To block the dectin-1 receptor, A549 cells were incubated in HBSS containing 5 µg/mL anti-dectin-1 mAb GE2 or isotype control antibody for 30 min. Thereafter, the swollen conidia or β-1,3-glucan were added to the cells at the indicated MOI for the PLD or the internalization assays.

### Flow cytometry

The cells were analyzed by flow cytometry for the expression of dectin-1 by using a FACS Calibur flow cytometer (Becton-Dickinson). The cells were incubated with primary anti-dectin-1 mAb GE2 (ab82888) for 30 min at 4°C (1∶1000), washed and treated with PE-conjugated anti-mouse IgG (1∶1000) for 30 min at 4°C. After washing twice with ice-cold PBS, the cells were immediately analyzed. Mouse IgG1 Ab was used as the isotype control antibody to assess the background staining. In each case, 30,000 cells were acquired. The data were analyzed using Cell Quest Pro (Becton-Dickinson).

### Statistical analysis

Data shown in the figures are either from a representative experiment or from mean ± SE of 3–4 independent experiments performed in triplicate. Except where otherwise indicated, Student's paired *t*-test was used to compare the difference between groups, and values of *P*<0.05 were considered statistically significant.
